# Concordance of Radiological, Laparoscopic and Laparotomic Scoring to Predict Complete Cytoreduction in Women with Advanced Ovarian Cancer

**DOI:** 10.3390/cancers15020500

**Published:** 2023-01-13

**Authors:** Mariano Catello Di Donna, Giuseppe Cucinella, Giulia Zaccaria, Giuseppe Lo Re, Agata Crapanzano, Sergio Salerno, Vincenzo Giallombardo, Giulio Sozzi, Anna Fagotti, Giovanni Scambia, Antonio Simone Laganà, Vito Chiantera

**Affiliations:** 1 Unit of Gynecologic Oncology, ARNAS “Civico-Di Cristina-Benfratelli”, 90127 Palermo, Italy; 2 Department of Surgical, Oncological and Oral Sciences (Di.Chir.On.S.), University of Palermo, 90133 Palermo, Italy; 3 Department of Biomedicine, Neuroscience and Advanced Diagnostics, University of Palermo, 90133 Palermo, Italy; 4 Gynecologic Oncology Unit, Department of Woman and Child Health and Public Health, Fondazione Policlinico Universitario A. Gemelli IRCCS, Largo Francesco Vito 1, 00168 Rome, Italy; 5 Università Cattolica del Sacro Cuore, Largo Francesco Vito 1, 00168 Rome, Italy; 6 Department of Health Promotion, Mother and Child Care, Internal Medicine and Medical Specialties (PROMISE), University of Palermo, 90133 Palermo, Italy

**Keywords:** ovarian cancer, cytoreductive surgery, prediction model, predictive index score, peritoneal cancer index score, primary debulking surgery, interval debulking surgery, neoadjuvant chemotherapy

## Abstract

**Simple Summary:**

In women affected by advanced ovarian cancer, complete cytoreductive surgery is of paramount important to achieve the best oncological outcomes. In this study, we compared radiologic, laparoscopic, and laparotomic scoring assessments to identify the best strategy to predict the achievement of complete cytoreductive surgery, both in upfront surgery and in neoadjuvant chemotherapy and subsequent surgery. We found that laparoscopic score assessment had a high accuracy for optimal cytoreduction in women affected by advanced ovarian cancer who need to undergo surgical management.

**Abstract:**

Objective: To identify the best method among the radiologic, laparoscopic and laparotomic scoring assessment to predict the outcomes of cytoreductive surgery in patients with advanced ovarian cancer (AOC). Methods: Patients with AOC who underwent pre-operative computed tomography (CT) scan, laparoscopic evaluation, and cytoreductive surgery between August 2016 and February 2021 were retrospectively reviewed. Predictive Index (PI) score and Peritoneal Cancer Index (PCI) scores were used to estimate the tumor load and predict the residual disease in the primary debulking surgery (PDS) and interval debulking surgery (IDS) after neoadjuvant chemotherapy (NACT) groups. Concordance percentages were calculated between the two scores. Results: Among 100 eligible patients, 69 underwent PDS, and 31 underwent NACT and IDS. Complete cytoreduction was achieved in 72.5% of patients in the PDS group and 77.4% in the IDS. In patients undergoing PDS, the laparoscopic PI and the laparotomic PCI had the best accuracies for complete cytoreduction (R0) [area under the curve (AUC) = 0.78 and AUC = 0.83, respectively]. In the IDS group, the laparotomic PI (AUC = 0.75) and the laparoscopic PCI (AUC= 0.87) were associated with the best accuracy in R0 prediction. Furthermore, radiological assessment, through PI and PCI, was associated with the worst accuracy in either PDS or IDS group (PI in PDS: AUC = 0.64; PCI in PDS: AUC = 0.64; PI in IDS: AUC = 0.46; PCI in IDS: AUC = 0.47). Conclusion: The laparoscopic score assessment had high accuracy for optimal cytoreduction in AOC patients undergoing PDS or IDS. Integrating diagnostic laparoscopy in the decision-making algorithm to accurately triage AOC patients to different treatment strategies seems necessary.

## 1. Introduction

Ovarian cancer is associated with the highest mortality rate among all cancers involving the female reproductive tract [1]. Approximately 70–80% of ovarian cancers are diagnosed at an advanced stage, with wide intraperitoneal dissemination and a subsequent poor prognosis (5-year survival rate: 20–30%) [2], especially after relapse [3,4]. Standard treatment for advanced ovarian cancer (AOC) is primary debulking surgery (PDS) followed by platinum-based chemotherapy [5,6,7]. The main goal of the surgical efforts is the removal of all macroscopically visible diseases. The presence of a residual tumor at the end of surgery is indeed recognized as the main negative prognostic factor for patients with AOC. In this context, complete cytoreductive surgery (R0) achieving no gross residual disease is associated with the best survival outcomes [8]. However, treatment of this condition often requires extensive multi-visceral surgery, with postoperative morbidity rates from 11.0 to 67.0% and mortality rates from 0 to 6.7% [9]. In selected patients who are not initially suitable for PDS due to comorbidities or a low likelihood of achieving optimal cytoreduction, neoadjuvant chemotherapy (NACT) followed by interval debulking surgery (IDS) represents a valid alternative therapeutic option [10,11].

Based on these elements, it is crucial to determine the best therapeutic approach between PDS (followed by adjuvant chemotherapy) or NACT (potentially followed by IDS in case of adequate response) at the time of diagnosis, in order to achieve complete removal of the disease and to minimize postoperative complications and therefore improve benefits of cytoreductive surgery.

Over the years, different score systems have been proposed and evaluated, aiming to assess the peritoneal spread of the disease and predict whether it is possible to obtain R0. Several imaging-based scoring models, using computed tomography (CT), have been suggested to predict the outcomes of PDS. These models included different radiological criteria, such as peritoneal thickening, ascites, para-aortic lymphadenopathy, and bowel involvement. Although the overall good predictive performance, the main limitation is represented by the unsuccessful rate when cross-validations datasets were used [12,13,14].

The Peritoneal Cancer Index (PCI), firstly described by Jacquet and Sugarbaker for mesothelioma and colon-rectal cancer [15], is a useful method to classify the degree of peritoneal carcinomatosis with a prognostic significance on AOC patients. However, this scoring procedure was initially validated for laparotomic abdominal exploration, with subsequent potential surgical risk and possible treatment delay [16].

Fagotti et al. [17] developed a laparoscopic scoring algorithm (predictive index, PI) including seven parameters based on intra-abdominal distribution of the disease. Although the accuracy of the laparoscopic model is 75% at predicting surgical outcome, the percentage of unnecessary laparotomies remains 33%, even after the inclusion of upper abdominal surgical score. Moreover, concordance between PI scores and PDS varies by anatomical location, with the lowest concordance in predicting bowel infiltration [18,19].

Although several models are validated and widely used in the clinical practice, there is a need to identify the system with the highest accuracy in predicting whether it is possible to obtain R0 and triage patients affected by AOC to alternative initial treatment strategies.

Considering this element, the aim of our study was to identify the best model (radiological vs. laparoscopic vs. laparotomic) to successfully predict the residual disease in AOC patients undergoing cytoreductive surgery. For this purpose, we compared the PI score and the PCI score determined at the time of preoperative CT scan, diagnostic laparoscopy, and laparotomic surgery, assessing the diagnostic accuracy to correctly triage patients to PDS or NACT.

## 2. Materials and Methods

### 2.1. Study Design and Enrollment

We performed a monocentric retrospective analysis of prospective-collected data of women affected by AOC treated at the Unit of Gynecologic Oncology, ARNAS “Civico-Di Cristina-Benfratelli” (Palermo, Italy) between August 2016 and February 2021, who met the following inclusion/exclusion criteria and signed informed consent for data collection and analysis for research purpose.

All patients aged ≥18 years with epithelial ovarian cancer in advanced stages (according to the International Federation of Gynecology and Obstetrics, FIGO 2018 stages ≥ IIIA) were considered eligible. Additional inclusion criteria were availability of pre-operative CT scan with contrast, curative-intent surgery, and Eastern Cooperative Oncology Group (ECOG) performance status ≤ 2 [20]. Exclusion criteria were histological diagnosis of ovarian cancer other than epithelial type, laparoscopic evaluation performed in other centers, unavailability of preoperative CT scan report, unknown residual tumor disease after cytoreductive surgery, or surgery performed only for symptom management (palliative surgery).

First, each woman underwent radiological evaluation with PI and PCI assessment, then diagnostic laparoscopy (with the only purpose of evalueting the extent and resectability of the disease) with PI and PCI assessment. After diagnostic laparoscopy, women were assigned to PDS or NACT, according to Institutional protocols based on the European Society for Medical Oncology (ESMO)—European Society of Gynecological Oncology (ESGO) consensus conference recommendations on ovarian cancer [21]. In case of women addressed to NACT (three to six cycles), a second radiological and laparoscopic assessment (using modified PI and PCI scoring according to Fagotti et al. and Sugarbacker et al. respectively [15,22]) was performed at the end of the chemotherapy. For the purpose of this data analysis, in case of NACT and subsequent IDS we considered and compared only the second radiologic and laparoscopic assessment.

The design, analysis, interpretation of data, drafting, and revisions were approved by the Institutional Review Board “Comitato Etico Palermo 2” (approval ID: 1047; date of approval: 19 December 2019), conform to the Helsinki Declaration, the Committee on Publication Ethics guidelines, and the Strengthening the Reporting of Observational Studies in Epidemiology Statement [23], available through the Enhancing the Quality and Transparency of Health Research Network. The data collected were anonymized, taking into account the observational nature of the study without personal data that could lead to formal identification of the patient. The study was not advertised. No remuneration was offered to the patients to be enrolled in this study.

### 2.2. Radiological, Laparoscopic and Laparotomic Evaluation

All patients included in the study underwent a pre-operative imaging staging, including CT scan of the chest, abdomen, and pelvis, as recommended by the European Society of Urogenital Radiology (ESUR) guidelines of 2010 [24].

During the CT scan-review, one single radiologist with more than 10 years of experience in gynecological oncology systematically assessed the radiological PI and PCI scores, for all the patients included in this analysis. The radiological PI was evaluated using the algorithm by Fagotti et al. [19,25], with 2 points assigned for each positive item, and a total score ranging from 0 (minimum) to 12 (maximum). Patients with mesenteric retraction were excluded from the current analysis, since infiltration of mesenteric root precludes optimal cytoreductive surgery with curative intent, for definition [26].

To quantify the radiological PCI, the abdomen was divided in 13 regions and in each one the maximum visible lesion size was measured and assigned to a lesion size (LS) score between 0 (minimum − no tumor seen) and 3 (maximum − tumor > 5 cm or confluence of more than one lesion), according to the scoring by Jacquet and Sugarbaker [16]. The sum of the score assigned to each region ranged between 1 and 39 points.

The PI and PCI scores were evaluated with the same criteria during diagnostic laparoscopy (laparoscopic PI and PCI) and cytoreductive surgery (laparotomic PI and PCI). All the diagnostic laparoscopies and cytoreductive surgeries were performed always by the same surgical team, with more than 10 years of experience in gynecological oncology for the management of AOC.

The comparison between radiological, laparoscopic, and laparotomic assessment was performed by subdividing women who underwent PDS and women who underwent IDS (in those cases, the comparison was done comparing radiological, laparoscopic and laparotomic assessment after NACT, in order to avoid any potential bias). In case of NACT, the PI was calculated by modified IDS score, according to Fagotti et al. [22] and further validation [27]. This score includes the evaluation of only 4 variables (mesenteric involvement, bowel involvement, gastric infiltration, liver surface involvement), with 2 points assigned for each positive items, and a total score ranging from 0 (minimum) to 8 (maximum). Similar to PDS analysis, we excluded women with mesenteric retraction post-NACT because infiltration of mesenteric root precludes optimal cytoreductive surgery for definition [26].

During cytoreductive surgery by laparotomic approach (either PDS or IDS), both PI and PCI were evaluated. The maximal surgical effort (achievement of ≤1 cm residual disease) was attempted in all patients and, when possible, included surgical removal of all tumor masses, along with total abdominal hysterectomy, bilateral salpingo-oophorectomy, radical omentectomy, appendectomy, multiple biopsies, and additional surgery (intestinal resections, total peritonectomy according to Sugarbaker’s technique, diaphragm stripping, diaphragmatic muscle resection, abdomino-pelvic peritoneal stripping, liver and pancreatic resection, splenectomy, cholecystectomy, aortic and/or pelvic lymphadenectomy) and/or hyperthermic intraperitoneal chemotherapy (HIPEC), if required.

Surgical complexity was classified using the Surgical Complexity Score system (SCS) described by Aletti et al. [28]. Surgical complexity was graded as low (≤3), intermediate (4–7), and high (≥8). Resection status and amount of residual disease following cytoreductive surgery was recorded for each patient. Complete cytoreductive surgery was defined as a no residual macroscopic tumor (R0), optimal cytoreductive surgery was defined as a residual tumor of ≤1 cm (R1), and suboptimal cytoreductive surgery was defined as a residual tumor of >1 cm (R2).

### 2.3. Statistical Analysis

Standard summary statistics were used to describe the demographic and clinical characteristics of the study population. Categorical data were compared with the χ^2^ test or Fisher’s exact test, as appropriate. The Mann–Whitney test was used to compare continuous variables. The agreement between radiologic assessment and laparoscopic and laparotomic evaluation based on the results of PI and PCI was analyzed graphically in the PDS and IDS group.

Sensitivity, specificity, positive predictive value (PPV), negative predictive value (NPV), agreement rate, and Cohen’s Kappa (95%CI) have been calculated for each parameter analyzed in the PI score. McNemar’s test was used to compare the two methods (CT scan and LPS).

The receiver operating characteristic curves (ROC curve) were developed comparing laparoscopic, radiologic, and laparotomic evaluation in order to select the best method to predict residual disease and the appropriate cut-off.

Univariate and multivariate analyses were used to detect the best test to predict R0 for both PDS and IDS group using the Cox regression models. Multivariate analysis was built modeling the R0 (yes/no) as the dependent variable and all possible factors which were found statistically significant (*p* < 0.05) at the univariate analysis as independent variables, calculating odds ratio (OR) and 95% confidence interval (CI).

All *p* values reported are two sided, and statistical significance was defined as *p* < 0.05. Statistical analysis was performed using the computer software SPSS version 26 (IBM SPSS Statistics for Windows, Version 26.0. Armonk, NY, USA).

## 3. Results

Our data analysis included 69 patients who underwent PDS, and 31 patients who underwent NACT followed by IDS. The demographic characteristics and clinical-pathologic data are reported in Table 1. We did not find significant differences between the group of women who underwent PDS and IDS for age (*p* = 0.22), Body Mass Index (*p* = 0.63), pre-operative CA 125 levels (*p* = 0.67), percentages of women affected by high grade serous ovarian cancer (HGSOC) and non-HGSOC (*p* = 0.06), SCS (*p* = 0.28), rates of R0, R1 and R2 after surgery (*p* = 0.51), rates of surgical procedure on the upper abdomen (*p* = 0.98), operative time (*p* = 0.74), estimated blood loss (*p* = 0.36), number of intraoperative transfusion (*p* = 0.7), intraoperative complication rate (*p* = 0.19), and length of hospital stay (*p* = 0.54) calculated from the hospital admission to discharge after final surgery.

### 3.1. Comparison of Radiological, Laparoscopic and Laparotomic Predictive Index (PI) and Peritoneal Cancer Index (PCI) in Women Who Underwent Primary Debulking Surgery

**Figure 1:**
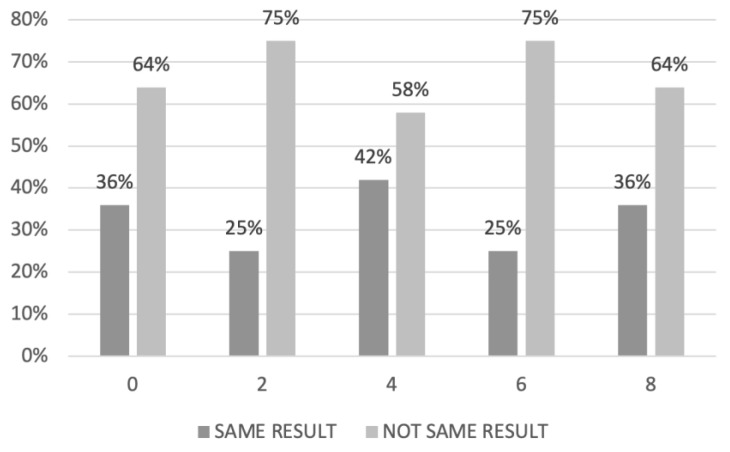
Agreement between laparoscopic and radiological predictive index (PI) in women who underwent primary debulking surgery.

In the PDS group, the highest concordance between laparoscopic and radiologic PI was found for a PI score of 4 (concordance rate: 42%). In contrast, the worst concordance was identified for PI value of 2 and 6 (Figure 1).

**Figure 2:**
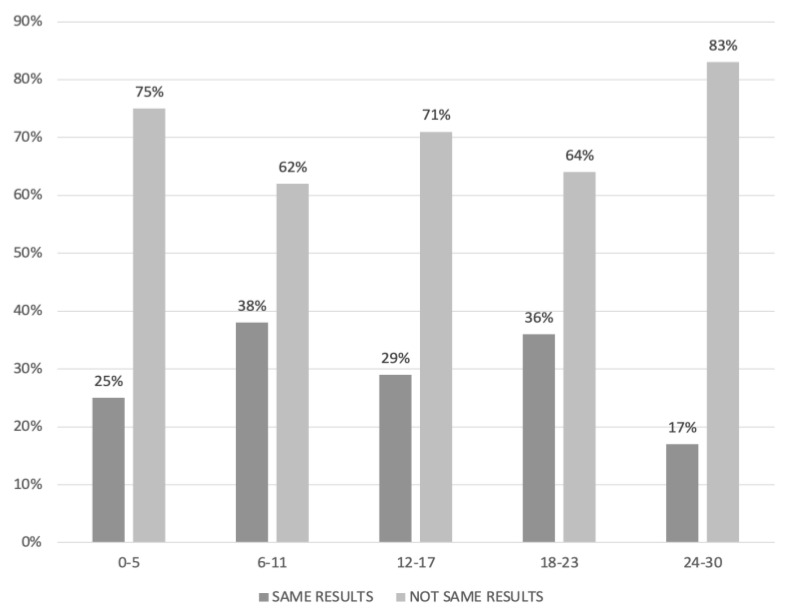
Agreement between laparotomic and radiological peritoneal cancer index (PCI) in women who underwent primary debulking surgery.

Regarding the agreement between the radiological and laparotomic PCI, range value of 6–11 was associated with the best concordance between the radiology and the surgery (concordance rate: 38%), while the worst correlation was observed for PCI values between 24 and 30 (Figure 2).

**Figure 3:**
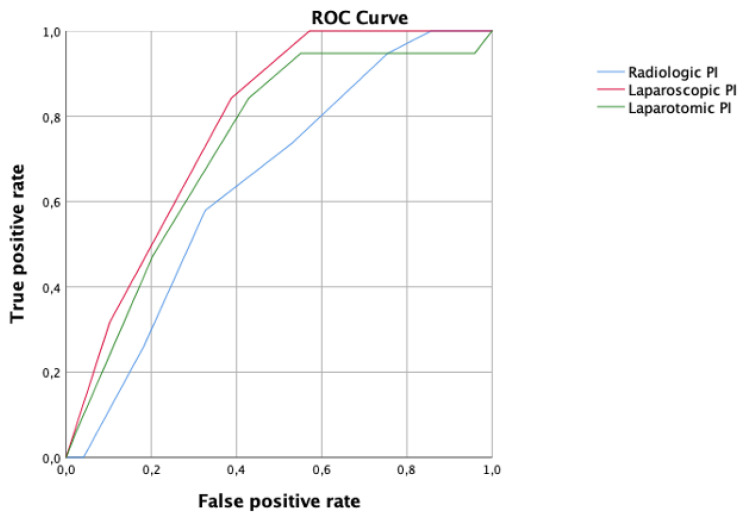
Receiver operating characteristic (ROC) curve comparing radiologic (blue line), laparoscopic (red line), and laparotomic (green line) predictive index (PI) in women who underwent primary debulking surgery.

In the PDS group, the best accuracy for the prediction of residual disease using the PI score was observed applying the laparoscopic PI with an AUC of 0.78, CI 95% 0.67–0.89. Additionally, the cut-off value associated with the best performance of the laparoscopic PI score was 6; conversely the accuracy of the radiological PI and laparotomic PI was AUC 0.64, CI 95% 0.50–0.78 and AUC 0.73, CI 95% 0.60–0.86, respectively (Figure 3).

Table 2 shows the concordance between PI scores at CT scan and laparoscopy, with the final findings at the PDS for each component of the score. In details, the radiological PI showed less accuracy than laparoscopy to discriminate the actual involvement of different items at time of PDS, specifically for the identification of peritoneal carcinomatosis (*p* = 0.027), diaphragmatic carcinomatosis (*p* < 0.01) and omental cake (*p* < 0.01).

**Figure 4:**
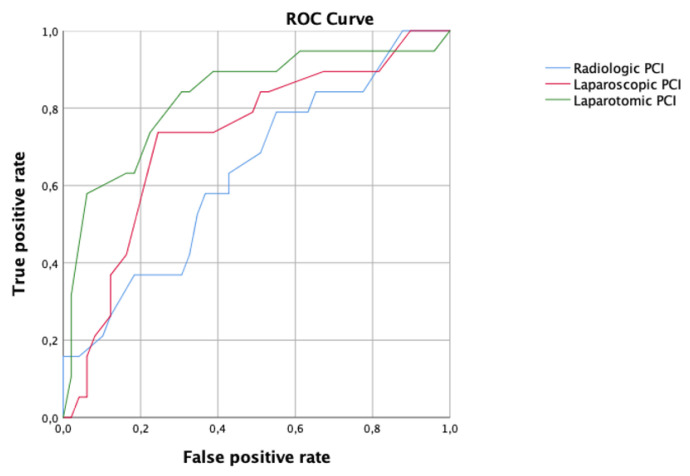
Receiver operating characteristic (ROC) curve comparing radiologic (blue line), laparoscopic (red line) and laparotomic (green line) peritoneal cancer index (PCI) in women who underwent primary debulking surgery.

Regarding the PCI score (Figure 4), the best performance to predict residual disease, with an AUC 0.83, CI 95% 0.71–0.95 was observed applying the laparotomic PCI, while the accuracy of the radiological PCI and laparoscopic PCI was AUC 0.64, CI 95% 0.49–0.78 and AUC 0.73, CI 95% 0.59–0.86, respectively. The cut-off value associated with the best performance of the laparotomic PCI score was 18.

On univariate analysis, the factors associated with the best prediction of R0 at the time of PDS were the laparoscopic PI (OR 1.72, CI 95% 1.24–2.39; *p* = 0.01), the radiologic PCI (OR 1.09, CI 95% 1.01–1.18; *p* = 0.04), and the laparotomic PCI (OR 1.23, CI 95%1.09–1.4; *p* = 0.001). Only the laparoscopic PI (OR 1.47, CI 95% 1.02–2.12, *p* = 0.04) was noted to be independent predictors of R0 on multivariate analysis (Table 3).

### 3.2. Comparison of Radiological, Laparoscopic and Laparotomic Predictive Index (PI) and Peritoneal Cancer Index (PCI) in Women Who Underwent Interval Debulking Surgery

**Figure 5:**
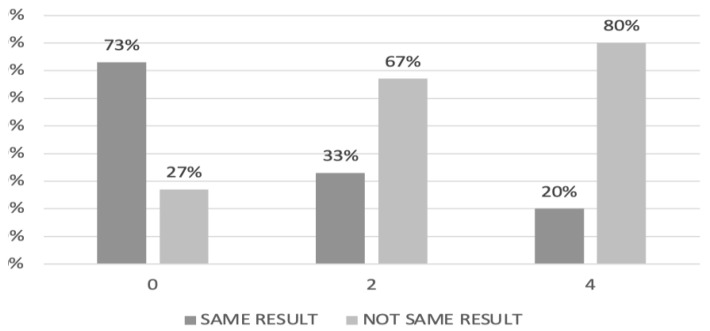
Agreement between laparoscopic and radiologic predictive index (PI) modified for interval debulking surgery (IDS).

In the IDS group, the highest concordance of the laparoscopic and radiological PI was observed with a PI score of 0 (concordance rate: 73%; Figure 5).

**Figure 6:**
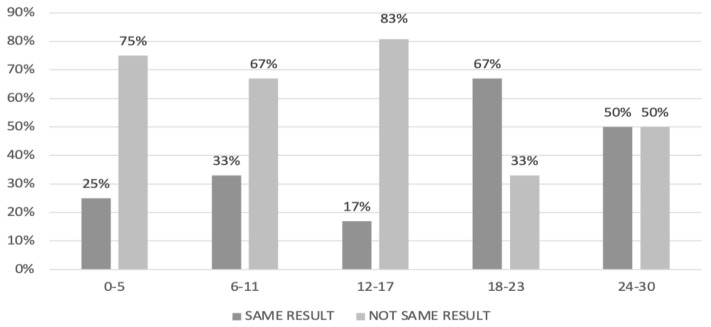
Agreement between laparotomic and radiologic peritoneal cancer index (PCI) in women undergoing interval debulking surgery.

The highest concordance of the laparotomic and radiological PCI was detected for values between 18 and 23 (concordance rate: 67%; Figure 6).

**Figure 7:**
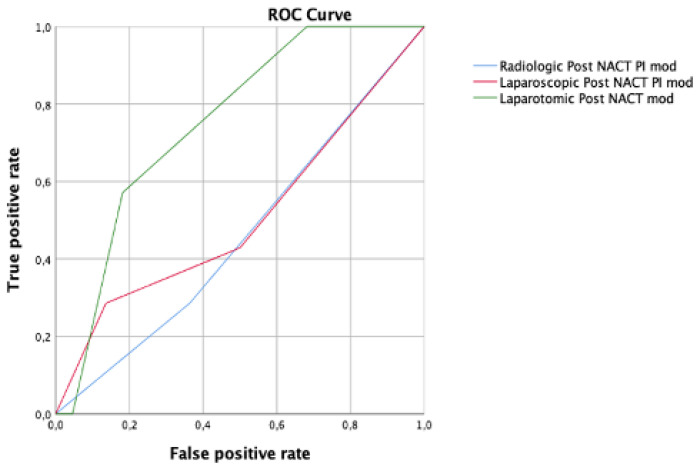
Receiver operating characteristic (ROC) curve comparing radiologic (blue line), laparoscopic (red line), and laparotomic (green line) predictive index (PI) modified for interval debulking surgery.

In the IDS group, the laparotomic PI score with a cut-off value of 4 represented the most accurate method to predict R0 at the time of IDS, with an AUC of 0.75 CI 95% 0.56–0.94. The accuracy of the radiological PI and laparoscopic PI was AUC 0.46, CI 95% 0.21–0.71 and AUC 0.51, CI 95% 0.24–0.77, respectively (Figure 7).

Specific concordance rates among modified PI scores at CT scan and laparoscopy, with the final findings at the IDS for each component of the score, are showed in Table 4.

**Figure 8:**
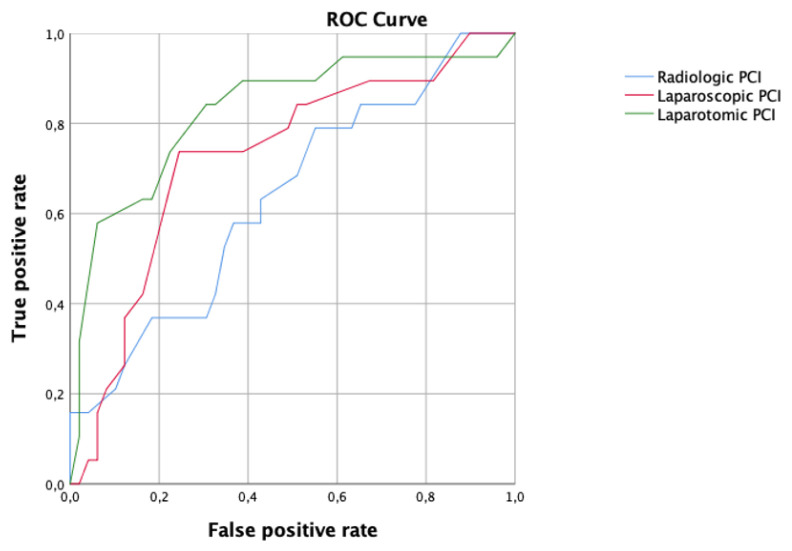
Receiver operating characteristic (ROC) curve comparing radiologic (blue line), laparoscopic (red line) and laparotomic (green line) peritoneal cancer index (PCI) in women who underwent interval debulking surgery.

Regarding the PCI score (Figure 8), the best performance to predict residual disease after NACT, with an AUC 0.87, CI 95% 0.73–1 was observed applying the laparoscopic PCI. In contrast, the accuracy of the radiological PCI and laparotomic PCI was AUC 0.47 CI 95% 0.17–0.77 and AUC 0.76 CI 95% 0.55–0.97, respectively. The cut-off value associated with the best performance of the laparoscopic PCI score was 19.

On univariate analysis (Table 5), the laparotomic PCI was the only factor associated with prediction of R0 at the time of IDS (OR 1.19, CI 95% 1.02–1.4; *p* = 0.02).

## 4. Discussion

In the current analysis, we identified the best method to predict the success of cytoreductive surgery, either as PDS or IDS, comparing PI and PCI values at CT scan, laparoscopy and laparotomy. Based on our results, the best method with the highest accuracy to predict the residual disease at the time of PDS is the laparotomic PCI (AUC = 0.83, CI 0.71–0.95). Regarding the setting of IDS, the laparoscopic PCI (AUC = 0.87, CI 0.73–1) had the best accuracy and represents the best method to predict residual disease outcome.

The best cut-off point associated with the most accurate method was 18 for the laparotomic PCI in the PDS group, and 19 for the laparoscopic PCI in the IDS group. In addition, the results showed the potential limitation of imaging when these specific scores are applied for the preoperative prediction of complete cytoreductive surgery, either in the PDS or IDS group. Indeed, the radiological PI and PCI were associated with the worst AUC when compared with the surgical evaluation (PI AUC = 0.64 and PCI AUC = 0.64 for PDS; PI AUC = 0.46 and PCI AUC = 0.47 for IDS).

Although a standardized method to help the clinician in the decision-making process between PDS and IDS for AOC patients is not established yet, our study confirmed that the use of the PI and PCI score is an acceptable and helpful tool, reinforcing data existing literature [17,19,22,25]. Nevertheless, there is still a need for a surgical invasive method to achieve an accurate triage result. Indeed, the transposition of PI and PCI scores during pre-operative CT scan failed in an accurate predictivity for both PDS and IDS.

Our results are in line with some previous studies on the role of CT imaging in the identification of abdominal disease in AOC. In these studies, the authors identified different radiologic predictors associated with suboptimal surgery. However, they were limited by the small sample size, inclusion of early stage disease, and variable rates of optimal cytoreduction (49% to 78%) [29,30,31].

Because of low detection rate in predicting no gross residual disease after surgery using CT images, some authors have introduced the use of laparoscopy in order to predict the resectability in patients with AOC. Fagotti et al. described a laparoscopic model based on a scoring system from 0 to a 12 according to 6 variables [25]; in the final analysis, this model identified SCS for scores ≥8 with a specificity of 100% (PPV 100%; NPV 70%). External validation of this score was performed by Brun et al. [32], who reported that laparoscopy-based score of ≥8 was associated with SCS, with sensitivity, specificity, PPV, NPV, and accuracy of 46%, 89%, 89%, 44%, and 60% respectively. Then, Petrillo et al. [17] identified a cut-off of 10 as laparoscopic PI as threshold to identify unfeasibility to achieve a complete cytoreduction. This result is probably due, at least in part, to the possibility to discriminate based on the difference between residual disease and post-chemotherapy scars through the laparotomic view.

Comparing CT evaluation and laparoscopic assessment with laparotomic exploration, our study identified laparoscopy as the best pre-operative tool for the definition of diaphragmatic, peritoneal, and omental disease spread, both in the PDS and IDS groups. The superiority of laparoscopy for omental cake evaluation could be explained with a different concept of omental disease between radiologic assessment and laparoscopic description. Moreover, the difference in the detection of peritoneal spread disease between the imaging and the laparoscopic camera could be explained with the very small size of the carcinomatosis with peritoneal and diaphragmatic implants. Indeed, very often diaphragmatic disease at laparoscopy shows as a liver’s metastasis on radiological imaging, due to the contact of the disease with liver’s surface.

In particular, after NACT, the CT scan does not seem accurate enough to define the exact extent of the disease for the potential reduction of the lesions or fails to recognize calcified residue after treatment. On the one hand, the need to integrate radiological evaluation with a surgical one could be considered even more evident in the specific subset of patients post-NACT. On the other hand, use of radiological evaluation alone may exclude patients who would instead be candidates for cytoreduction through surgical evaluation.

Despite the low accuracy of CT scan in the prediction of residual disease, the preoperative imaging maintains an important diagnostic role in the evaluation of tumor spread in anatomical sites not explorable with laparoscopy (such as the retro-cavity of the epiploon, the retro-hepatic area and the hepatic pedicle), and in the diagnosis of parenchymal and lymphatic metastasis. Extensive involvement of these anatomical areas can be a clear limit to complete cytoreduction. Moreover, previous studies have investigated the PCI score evaluated using 18F-fluorodeoxyglucose positron emission tomography/computed tomography (18F-FDG PET/CT), although regions corresponding to the small bowel have the lowest accuracy [33]. Laparoscopy, conversely, allows a direct visualization of the small bowel surface and the identification of diffuse carcinosis at this level. Furthermore, the use of 18F-FDG PET/magnetic resonance imaging (18F-FDG PET/MRI) has been proposed as a novel approach for estimating the extent of peritoneal carcinomatosis [34]. A recent study by Jónsdóttir et al. [35] compared the accuracy of PET/MRI with diffusion-weighted (DW)-MRI for predicting peritoneal carcinomatosis; the authors reported that PET/MRI was more accurate than DW-MRI when evaluating patients at primary diagnosis, but no difference was noted in patients after NACT.

Indeed, a multimodal approach is therefore crucial for the pre-operative evaluation of AOC patients, to overcome the limits of each technique.

More interesting data emerged from our analysis of the high accuracy of laparoscopic PCI in predicting the cytoreducibility in the context of patients who are candidates for IDS. On the one hand, the modified laparoscopic PI described by Fagotti et al. [22] analyzes four variables (mesenteric retraction, bowel and stomach infiltration, and superficial liver metastases), which are associated with high rate of suboptimal cytoreduction. On the other hand, laparoscopic PCI considers a broader assessment of all abdominal quadrants, besides to identify parameter for suboptimal cytoreduction.

Overall, our findings emphasize that surgical evaluation (by laparoscopy and laparotomy) and a consequent score’s attribution seems to be the most appropriate method to predict which patients would have more benefit from IDS or PDS. In this scenario, diagnostic laparoscopy with the validated PI score allows an evaluation of the tumor load in order to plan upfront surgery or NACT according to the patient’s clinical characteristics, avoiding the risk of unnecessary laparotomic exploration.

Given the multifactorial assessment required for the correct evaluation of AOC patients, a predictive model aimed to integrate clinical or surgical data could be helpful. Based on these elements, Piedimonte et al. [36] developed a 4-step prediction model for outcome of optimal cytoreduction at PDS. Integrating clinical, surgical, and radiological parameters (unresectables stage IVb, patient factors, surgical resectability scores, and SCS) the model reached 85% sensitivity, 75% specificity, and 85% accuracy. In this cohort of 185 AOC patients, the use of this algorithm would have correctly triaged 3 suboptimally cytoreduced patients to receive NACT.

Moreover, a novel strategy to improve preoperative prediction of surgical outcome was recently suggested by the Mayo Clinic group [37]. Preoperative CT imaging combined with tumor molecular subtyping (mesenchymal subtype or not) helped to identify AOC patients more likely to have high complexity surgery. Women with higher CT score and mesenchymal subtype were associated with very low likelihood of complete cytoreduction (OR = 26.73, 95% CI = [6.42, 186.94]; *p* < 0.001).

In our data analysis, despite the complexity of the surgery being high (SCS 3 in the 68% of cases), 74 patients underwent optimal cytoreductive surgery with no residual disease, R0. However, these data are limited to a restricted patient population treated by the same surgical team. In addition, we take the opportunity to remark that in our setting laparoscopy was used with the only purpose of assessing the extent and resectability of the disease in order to decide between PDS or NACT followed by IDS, and this correlates with the lack of laparoconversion in this series. Both PDS and IDS were started with laparotomic approach.

Regarding the role of minimally invasive surgery (MIS) in ovarian cancer, our report highlights the fundamental role of laparoscopy in diagnosing the extensiveness of ovarian cancer in case of advanced disease. However, recent evidence introduced robotic surgery for interval debulking after NACT in selected patients showing similar surgical and oncological outcomes as in open surgery [38]. Although the robotic approach may represent an evolution of laparoscopy, especially in the case of surgery on morbidly obese patients, some authors suggest that MIS should be limited to standard cytoreductive procedures of low complexity [39]. In this regard, performing a laparoscopy after NACT and before IDS to exclude peritoneal disease and/or patients requiring additional complex surgical procedures could maximize the benefits of robotic surgery [40,41].

To the best of our knowledge, this is the first report comparing radiology, laparoscopy, and laparotomy, according to validated scores based on the likelihood of achieving optimal cytoreduction. The strength of our study lies in the direct and systematic comparison of the radiological, laparoscopic, and laparotomic scoring assessments for AOC. Moreover, the pool of complex surgical cases is described in a homogenous cohort of patients with primary epithelial ovarian cancer treated with high rate of R0 in a tertiary care center by the same surgical team, with long-term experience for the management of AOC. Another strength is the review of all radiological images by one radiologist expert about gynecologic oncology. Compared to the literature, this study also included the radiological and laparotomic PI in the tumor load evaluation of patients with AOC. Additionally, our study also extended the analysis to patients undergoing NACT-IDS. The inclusion of patients who underwent IDS allows to identify those with low likelihood of achieving R0, driving the decision to further cycles of chemotherapy or switch to different lines of chemotherapy.

The main limitations of our study are the retrospective nature of the investigation and the relatively small sample size. However, because of the important impact of residual disease in prognosis of AOC, our study could be of clinical interest to reduce the number of unnecessary laparotomic explorations and to select the best patients that could benefit from NACT, and moreover to increase the number of IDS who otherwise, based on a single radiological evaluation, without an accurate surgical exploration, would be candidates for further chemotherapy treatment, losing a valid chance of cure. From this perspective, a validation of the models in large cohort analysis, suing prospective approach, remains necessary. In addition, in this study we could not provide a direct comparison of disease-free survival in laparoscopy versus laparotomy: indeed, laparoscopy was performed with the only purpose of diagnosis the extent and resectability of the disease, and then all the cytoreductive surgeries (both PDS and IDS) were performed by laparotomic approach.

Further steps should be done to identify novel radiological variables to increase the accuracy of the preoperative CT scan in the prediction of R0. In addition, either the role of radiological or laparoscopic assessment in the recurrence cases should be explored to predict the feasibility of secondary cytoreductive surgery. Indeed, besides the validated criteria, such as the Arbeitsgemeinschaft Gynäkologische Onkologie (AGO) and Memorial Sloan Kettering (MSK), in predicting a complete secondary cytoreductive surgery, the question regarding the selection of the patient who could benefit more from the surgical treatment is still open [42]. Indeed, considering radiological and laparoscopic scores during the decisional algorithm could help to clarify this question in the future.

Future research may aim to integrate preoperative score with biological features of the tumor, even in the setting of machine learning algorithm(s). The artificial intelligence technology may assist in the triage between NACT and PDS and can be enriched by specific panel gene testing to improve the accuracy of an ideal integrated predictive model.

## 5. Conclusions

Our data analysis suggests a scarce predictivity of the CT scan alone in the prediction of residual disease in AOC patients who underwent PDS or IDS. The laparoscopic intraoperative assessment of the tumor load with the use of validated score (PI for PDS and PCI for IDS) represents a valuable method with high accuracy to identify the best candidate for a successful cytoreductive surgery.

## Figures and Tables

**Table 1:** Demographic characteristics and clinical-pathologic data. PDS: primary debulking surgery; IDS: interval debulking surgery; FIGO: International Federation of Gynecology and Obstetrics; HGSOC: high grade serous ovarian cancer; EBL: estimated blood loss.

Variables	Total	PDS	IDS	*p* *
Age years, median (range)	59.6 (28–90)	59 (35–90)	61 (28–80)	0.22
Body Mass Index, median (range)	26.2 (17–51)	26.2 (17–51)	26.3 (18–44)	0.63
Pre-operative CA 125 (UI/mL)				
Negative, n (%)	7 (7.1%)	7 (10.4%)	0 (0%)	0.67
<500 UI/mL, n (%)	35 (35.7%)	23 (34.3%)	12 (38.7%)	
≥500 UI/mL, n (%)	56 (57.1%)	37 (55.2%)	19 (61.3%)	
FIGO stage				
IIIA, n (%)	9 (9%)	9 (13%)	0 (0%)	0.001
IIIB, n (%)	5 (5%)	5 (7.2%)	0 (0%)	
IIIC, n (%)	61 (61%)	47 (68.2%)	17 (54.8%)	
IVA, n (%)	5 (5%)	2 (2.9%)	3 (9.7%)	
IVB, n (%)	17 (17%)	6 (8.7%)	11 (35.5%)	
Histology				
HGSOC, n (%)	83 (83%)	54 (78.3%)	29 (93.5%)	0.06
Non-HGSOC, n (%)	17 (17%)	15 (21.7%)	2 (6.5%)	
Surgical complexity score				
1, n (%)	11 (11%)	6 (8.7%)	5 (16.1%)	0.28
2, n (%)	21 (21%)	14 (20.3%)	7 (22.6%)	
3, n (%)	68 (68%)	49 (71%)	19 (61.3%)	
Residual Disease				
R0, n (%)	74 (74%)	50 (72.5%)	24 (77.4%)	0.51
R1 (0.1–1 cm), n (%)	22 (22%)	15 (21.7%)	7 (22.6%)	
R2 (>1 cm), n (%)	4 (4%)	4 (5.8%)	0 (0%)	
Upper abdomen procedures (UAP)				
Yes	84 (84%)	58 (84.1%)	26 (83.9%)	0.98
No	16 (16%)	11 (15.9%)	5 (16.1%)	
Operative time (min), median (range)	422.5 (90–870)	421.5 (90–870)	424.7 (170–700)	0.74
EBL (mL), median (range)	428.9 (50–2000)	460.9 (50–2000)	362.9 (50–900)	0.36
Number of intra-operative transfusion (n), median (range)	0.4 (0–1)	0.4 (0–1)	0.5 (0–1)	0.7
Length of hospital stay (days **), median (range)	17.4 (0–68)	18 (0–68)	16.1 (3–60)	0.54

* Categorical data were compared with the χ^2^ test or Fisher’s exact test, as appropriate. The Mann–Whitney test was used to compare continuous variables. ** Calculated from the hospital admission to discharge after final surgery.

**Table 2:** Concordance between predictive index (PI) scores at computed tomography (CT) scan and laparoscopy (LPS) in women who underwent primary debulking surgery. PPV: positive predictive value; NPV: negative predictive value.

Variables	Method	Sensitivity (%)	Specificity (%)	PPV (%)	NPV (%)	Agreement (%)	Cohen’s Kappa (95%CI)	*p* *
Peritoneal carcinomatosis	CT scan	95%	27%	72%	75%	72%	0.27 (95%CI −0.02–0.56)	0.027
	LPS	88%	86%	93%	79%	88%	0.73 (95%CI 0.56–0.91)	
Diaphragmatic carcinomatosis	CT scan	60%	76%	88%	41%	65%	0.29 (95%CI 0.05–0.52)	<0.01
	LPS	92%	94%	98%	80%	92%	0.81 (95%CI 0.65–0.97)	
Omental cake	CT scan	67%	68%	81%	52%	68%	0.33 (95%CI 0.09–0.57)	<0.01
	LPS	86%	95%	97%	78%	89%	0.77 (95%CI 0.61–0.93)	
Stomach, and/or lesser omentum, and/or spleen	CT scan	43%	72%	71%	44%	54%	0.13 (95%CI −0.1–0.36)	0.84
	LPS	18%	100%	100%	43%	49%	0.14 (95%CI −0.07–0.35)	
Bowel involvement	CT scan	54%	62%	52%	64%	58%	0.16 (95%CI −0.09–0.4)	0.2
	LPS	36%	95%	83%	66%	69%	0.33 (95%CI 0.08–0.57)	
Liver surface lesions >2 cm	CT scan	57%	86%	33%	94%	83%	0.33 (95%CI −0.03–0.69)	0.23
	LPS	14%	100%	100%	91%	91%	0.23 (95%CI −0.36–0.82)	

* McNemar’s test was used to compare the two methods (CT scan and LPS).

**Table 3:** Univariate and multivariate analyses of the factors predicting complete cytoreduction (R0) at the time of primary debulking surgery. PI: predictive index; PCI: peritoneal cancer index; OR: odds ratio; CI: confidence interval.

	Univariate Analysis		Multivariate Analysis	
	OR (CI 95%)	*p* *	OR (CI 95%)	*p* *
Age	1.03 (0.98–1.08)	0.29	/	/
Body mass index	1.04 (0.97–1.13)	0.26	/	/
Previous surgery (no vs. yes)	1.41 (0.49–4.08)	0.52	/	/
CA 125	1 (0.99–1)	0.45	/	/
Radiologic PI	1.18 (0.99–1.42)	0.64	/	/
Radiologic PCI	1.09 (1.01–1.18)	0.04	1.09 (0.97–1.22)	0.15
Laparoscopic PI	1.72 (1.24–2.39)	0.01	1.47 (1.02–2.12)	0.04
Laparotomic PCI	1.23 (1.09–1.4)	0.001	1.13 (0.99–1.30)	0.07

* Univariate and multivariate analysis were used to detect the best test to predict R0, using the Cox regression models.

**Table 4:** Concordance between modified predictive index (PI) scores at computed tomography (CT) scan and laparoscopy (LPS) in women who underwent interval debulking surgery. PPV: positive predictive value; NPV: negative predictive value; CI: confidence interval.

Variables	Method	Sensitivity (%)	Specificity (%)	PPV (%)	NPV (%)	Agreement (%)	Cohen’s Kappa (95%CI)	*p* *
Stomach, and/or lesser omentum, and/or spleen	CT scan	35%	70%	70%	35%	47%	0.04 (95%CI −0.28–0.36)	0.58
	LPS	37%	89%	88%	40%	54%	0.19 (95%CI −0.13–0.52)	
Bowel involvement	CT scan	29%	70%	22%	76%	60%	−0.02 (95%CI −0.46–0.43)	0.12
	LPS	71%	86%	63%	90%	82%	0.55 (95%CI 0.18–0.91)	
Liver surface lesions >2 cm	CT scan	20%	96%	50%	86%	83%	0.21 (95%CI −0.42–0.84)	1
	LPS	40%	91%	50%	88%	82%	0.34 (95%CI −0.19–0.86)	

* McNemar’s test was used to compare the two methods (CT scan and LPS).

**Table 5:** Univariate analysis of the factors predicting complete cytoreduction (R0) at the time of primary debulking surgery. NACT: neoadjuvant chemotherapy; PI: predictive index; PCI: peritoneal cancer index; OR: odds ratio; CI: confidence interval.

	Univariate Analysis	
	OR (CI 95%)	*p* *
Age	1.02 (0.95–1.11)	0.56
Body mass index	1.12 (0.95–1.32)	0.18
Previous surgery (no vs. yes)	0.67 (0.12–3.73)	0.64
CA 125	1 (0.99–1)	0.72
Radiologic post-NACT PI (modified)	0.83 (0.47–1.45)	0.51
Radiologic post-NACT PCI	0.96 (0.85–1.1)	0.56
Laparoscopic post-NACT PI (modified)	1.1 (0.61–1.86)	0.81
Laparotomic post-NACT PCI	1.19 (1.02–1.4)	0.02

* Univariate analysis was used to detect the best test to predict R0, using the Cox regression models.

## Data Availability

Full dataset will be available from the first author (M.C.D.D.) on reasonable request.
